# *Lactobacillus casei-01* Facilitates the Ameliorative Effects of Proanthocyanidins Extracted from Lotus Seedpod on Learning and Memory Impairment in Scopolamine-Induced Amnesia Mice

**DOI:** 10.1371/journal.pone.0112773

**Published:** 2014-11-14

**Authors:** Juan Xiao, Shuyi Li, Yong Sui, Qian Wu, Xiaopeng Li, Bijun Xie, Mingwei Zhang, Zhida Sun

**Affiliations:** 1 College of Food Science and Technology, Huazhong Agricultural University, 1 Shizishan Street, Hongshan District, Wuhan 430070, Hubei, China; 2 Key Laboratory of Functional Foods, Ministry of Agriculture/ Guangdong Key Laboratory of Agricultural Products Processing, Sericultural & Agri-Food Research Institute Guangdong Academy of Agricultural Sciences, 133 Yiheng Road, Dongguan Zhuang, Tianhe District, Guangzhou 510610, Guangdong, China; 3 College of Food Science and Engineering, Wuhan Polytechnic University, Xuefu South Road, Changqing Garden, Dongxihu District, Wuhan 430023, Hubei, China; Jilin University, China

## Abstract

Learning and memory abilities are associated with alterations in gut function. The two-way proanthocyanidins-microbiota interaction *in vivo* enhances the physiological activities of proanthocyanidins and promotes the regulation of gut function. Proanthocyanidins extracted from lotus seedpod (LSPC) have shown the memory-enhancing ability. However, there has been no literature about whether *Lactobacillus casei-01* (LC) enhances the ameliorative effects of LSPC on learning and memory abilities. In this study, learning and memory abilities of scopolamine-induced amnesia mice were evaluated by Y-maze test after 20-day administration of LC (10^9^ cfu/kg body weight (BW)), LSPC (low dose was 60 mg/kg BW (L-LSPC) and high dose was 90 mg/kg BW (H-LSPC)), or LSPC and LC combinations (L-LSPC+LC and H-LSPC+LC). Alterations in antioxidant defense ability and oxidative damage of brain, serum and colon, and brain cholinergic system were investigated as the possible mechanisms. As a result, the error times of H-LSPC+LC group were reduced by 41.59% and 68.75% relative to those of H-LSPC and LC groups respectively. LSPC and LC combinations ameliorated scopolamine-induced memory impairment by improving total antioxidant capacity (TAOC) level, glutathione peroxidase (GSH-Px) and total superoxide dismutase (T-SOD) activities of brain, serum and colon, suppressing malondialdehyde (MDA) level of brain, serum and colon, and inhibiting brain acetylcholinesterase (AchE), myeloperoxidase, total nitric oxide synthase and neural nitric oxide synthase (nNOS) activities, and nNOS mRNA level. Moreover, LC facilitated the ameliorative effects of H-LSPC on GSH-Px activity of colon, TAOC level, GSH-Px activity and ratio of T-SOD to MDA of brain and serum, and the inhibitory effects of H-LSPC on serum MDA level, brain nNOS mRNA level and AchE activity. These results indicated that LC promoted the memory-enhancing effect of LSPC in scopolamine-induced amnesia mice.

## Introduction

Accumulating data indicate that imbalance of gut bacteria not only contributes to gut dysfunction [1], chronic metabolic disorders [2] and aging [3], but also plays an important role in memory and cognition dysfunction [1], [4]. Recent researches have reported that learning and memory abilities in mice are associated with diet-induced alterations in gut bacteria [1], [5]. *C rodentium* infection or high-fat diet resulted in memory impairment via disturbing the balance of gut bacteria, which were reversed by regulation of gut microbiota and colonic inflammatory [1], [6]. Moreover, McCarthy investigated the concomitant symptoms of dementia patients randomly in 20 health districts of England, and found that 85% of the patients suffered from gut dysfunction [7]. Relieving symptoms of gut dysfunction such as constipation was beneficial to the attenuation of memory and cognition dysfunction in dementia patients [8]. Therefore, modulation of gut microbiota and gut function may be the potential strategies for reversing learning and memory deficits and related diseases [9]–[11].

Lotus seedpod, a part of *Nelumbo nucifera* Gaertn, contains an abundance of proanthocyanidins. It has been demonstrated that the main components in LSPC are monomers, dimers, trimers and tetramers of proanthocyanidins, in which dimers are the most component, and catechin and epicatechin are the base units [12]. LSPC have been proven to possess a potent antioxidant activity [12], and ameliorate memory deficits and oxidative damage in scopolamine-induced amnesia mice, SAMP8 and cognitively impaired aged rats [13]–[15]. However, there is no literature about the gut-regulation effect of LSPC. Previous studies have reported that proanthocyanidins B1 and B2, and proanthocyanidins in red wine modulate intestinal function by regulation of microbiota [16], [17]. Moreover, the oligomer and polymer of proanthocyanidins are poorly absorbed compared to the monomers in small intestine, and then accumulate to colon, where they are metabolized by the microbiota to more bioactive compounds with potent physiological effects [18], [19]. Importantly, proanthocyanidins and their metabolites in colon play an important role in the maintenance of intestinal health by inhibiting the growth of pathogenic bacteria, and keeping the growth of probiotics such as *Lactobacillus* [20]. Thus, the gut-modulation effect of B-type proanthocyanidins and the two-way proanthocyanidins-microbiota interaction suggest that LSPC may have a gut-modulation effect.

It is generally accepted that probiotics contribute to the balance of gut microecology and gut health [1], [6]. Recently, accumulating evidence has revealed the memory-enhancing effect of probiotics. Davari and colleagues have reported that administration of mixed probiotics pronouncedly ameliorates spatial memory impairment in diabetic rats by recovering basic synaptic transmission and hippocampal long-term potentiation, and inhibiting oxidative damage [21]. *Lactobacillus pentosus var. plantarum* C29 attenuates scopolamine-induced amnesia by inducing brain BDNF and p-CREB expressions [22]. Moreover, several researches have shown that probiotics prevent memory deficits by modulating gut function. The study of Gareau and colleagues has revealed that combination of *Lactobacillus rhamnosus* and *Lactobacillus helveticus* improves spatial memory impairment in *C rodentium*-infected mice by regulation of gut microbiota and inhibition of colonic inflammatory and epithelial cell hyperplasia [1]. Ameliorative effects of *Lactobacillus helveticus* on memory deficits in IL-10^−/−^ mice with high-fat diet are closely associated with the modulation of gut microbiota, colonic inflammation and cytokine expression [6].

It has been proven that both probiotics and LSPC have the memory-enhancing effect and gut-modulation effect. And there is a two-way interaction between probiotics and LSPC [19], [20]. However, there has been no literature about whether probiotics enhance the ameliorative effects of LSPC on learning and memory ability. Our lab found that oligomeric proanthocyanidins from Litchi pericarp did not change the growth of *Lactobacillus casei-01* at concentrations of 0.25 and 0.5 mg/mL *in vitro*, and were decomposed into many kinds of phenolic acids with more potent antioxidant ability than their parent proanthocyanidins [23]. These results indicate that *Lactobacillus casei-01 in vivo* may enhance the biotransformation of LSPC, and that may further increase the ameliorative effects of LSPC on intestinal function and learning and memory ability.

The purpose of this study was to investigate whether *Lactobacillus casei-01* enhanced the ameliorative effects of LSPC on learning and memory ability in scopolamine-induced amnesia mice. Alterations in antioxidant defense ability and oxidative damage of brain, serum and colon, and brain cholinergic system were investigated as the possible mechanisms.

## Materials and Methods

### Chemical and reagents

Mature lotus seedpods of *Nelumbo Nucifera* Gaertn (cultivar: Number 2 Wuhan plant) were harvested from Honghu District (113°7′–114°5′E, 29°39′–30°2′N) in Hubei province, China, in late July, 2011, and arrived in the laboratory within 24 h postharvest, which were kept at −20°C prior to extraction. Lotus seedpod was identified by Prof. Xueming Ni from Department of Botany, Wuhan Plant Institute of the Chinese Academy of Science. *Lactobacillus casei-01* was purchased from Chr. Hansen Company (Beijing, China).

Kits for determination of glutathione peroxidase (GSH-Px, Cat. No. A005), total superoxide dismutase (T-SOD, Cat. No. A001-1), acetylcholinesterase (AchE, Cat. No. A024), total nitric oxide synthase (TNOS, Cat. No. A014-1), neural nitric oxide synthase (nNOS, Cat. No. A014-1), inducible nitric oxide synthase (iNOS, Cat. No. A014-1) and myeloperoxidase (MPO, Cat. No. A044) activities, levels of malondialdehyde (MDA, Cat. No. A003-1), total antioxidant capacity (TAOC, Cat. No. A015) and protein (Cat. No. A045-2) were purchased from Nianjing Jiancheng Bioengineering Institute, Nanjing, China. Scopolamine hydrobromide injection was purchased from Xuzhou RYEN Pharma.CO., LTD, Xuzhou, China. Piracetam was purchased from Hubei Huazhong Pharma.CO., LTD, Hubei, China.

### Preparation of LSPC and strain

Frozen lotus seedpods were extracted to obtain LSPC as described previous [24]. LSPC were stored at −20°C and the purity of 98.7% was measured on the basis of comparison with a calibration curve of grape seed procyanidin extract by the method reported by Porter [25]. Electrospray ionization mass spectrometry analysis has revealed that monomers, dimers, trimers and tetramers of proanthocyanidins are the main ingredients of LSPC, and the base units of LSPC are (+)-catechin and (−)-epicatechin [12]. The percentage of catechin, epicatechin, dimers, trimers and tetramers in LSPC is 10.9%, 9.1%, 53.6%, 19.5% and 1.9%, respectively. *Lactobacillus casei-01* powder was incubated in MRS fluid nutrient medium at 37°C for 18 h to activate. Then, the viable bacteria were inoculated (1%) in the same medium, which was incubated at 37°C for 24 h to reach the strain concentration at about 10^7^ cfu/mL. The strain solution (50 mL) was put in a centrifuge tube, and centrifuged at 5590 *g* for 10 min at 4°C. The precipitate was dissolved in distilled water (50 mL) and centrifuged at 5590 *g* for 10 min at 4°C, which was repeated two times to remove the MRS. The precipitate was the strain (5×10^8^ cfu in each tube) and stored at 4°C.

### Animals

Male Kunming mice (20±2 g) were purchased from Wuhan University Research Center for Animal Experiment, China. Animals were housed five per cage, kept under a controlled temperature 22±1°C, humidity 55–60% and 12-h light/12-h dark cycle throughout the experiment. A normal solid diet and water were available *ad libitum*. The diet was provided by the Wuhan University Research Center for Animal Experiment, China.

### Ethics statement

All experimental procedures involving animals followed the Guiding Principles in the Care and Use of Animals, and were approved by the ethics committee of Wuhan General Hospital of Guangzhou Military Command (SYXK (Hubei) 2008-0007), and Huazhong Agricultural University, Hubei Province, China. We made all efforts to minimize suffering. The animals were killed by cervical dislocation under anesthesia. Mature lotus seedpods used in this study were industrial crops and obtained from private land, for future permissions should be contacted with Zong Zhang (+86 13972369298). No specific permissions were required for Honghu District, Hubei province, China. The study did not involve any endangered or protected species.

### Treatments

After acclimatization for 3 days, the animals were chosen by Y-maze test. Then, a total of 80 male Kunming mice were randomly divided into eight groups with ten mice in each group: control (CON), vehicle scopolamine control (SCOP), positive drug control (Piracetam), *Lactobacillus casei-01* (LC), low and high dose of LSPC (L-LSPC and H-LSPC), L-LSPC and LC combination (L-LSPC+LC), H-LSPC and LC combination (H-LSPC+LC) groups (Table 1). They were given the respective drugs at a dose of 0.1 mL/10 g body weight (BW) by oral gavage once daily for 20 days. Groups of CON, SCOP, Piracetam, LC, L-LSPC and H-LSPC were given distilled water, distilled water, piracetam (400 mg/kg BW), LC (10^9^ cfu/kg BW), LSPC (60 mg/kg BW) and LSPC (90 mg/kg BW), respectively. L-LSPC+LC and H-LSPC+LC were given a mixture of LSPC (60 mg/kg BW) and LC (10^9^ cfu/kg BW), and a mixture of LSPC (90 mg/kg BW) and LC (10^9^ cfu/kg BW), respectively (Table 1).

**Table 1:** Groups and treatments.

Groups (abbreviations)	Treatments^a^ (oral gavage, 1^st^–20^th^ day)	Induce memory impairment^b^ (intraperitoneal, the 20^th^ day)
Control (CON)	Distilled water	Saline
Vehicle scopolamine control (SCOP)	Distilled water	3 mg/kg BW scopolamine
Positive drug control (Piracetam)	400 mg/kg BW^c^ Piracetam	3 mg/kg BW scopolamine
*Lactobacillus casei-01* (LC)	10^9^ cfu/kg BW LC	3 mg/kg BW scopolamine
Low dose of LSPC (L-LSPC)	60 mg/kg BW LSPC	3 mg/kg BW scopolamine
High dose of LSPC (H-LSPC)	90 mg/kg BW LSPC	3 mg/kg BW scopolamine
L-LSPC and LC combination (L-LSPC+LC)	60 mg/kg BW LSPC and 10^9^ cfu/kg BW LC combination	3 mg/kg BW scopolamine
H-LSPC and LC combination (H-LSPC+LC)	90 mg/kg BW LSPC and 10^9^ cfu/kg BW LC combination	3 mg/kg BW scopolamine

a : Administrated once daily by oral gavage from 1^st^–20^th^ day;

b : Administrated intraperitoneal 30 min before a training course of Y-maze test at the 20^th^ day;

c : body weight.

Solutions of Piracetam (40 mg/mL), L-LSPC (6 mg/mL LSPC) and H-LSPC (9 mg/mL LSPC) were prepared in distilled water. Five mL of distilled water, L-LSPC and H-LSPC solutions were mixed with the strains in three tubes, respectively, as the solutions for the groups of LC, L-LSPC+LC and H-LSPC+LC. Doses of LC, LSPC, and LSPC and LC combinations were based on the early experiments results *in vivo* and in *vitro*. All solutions were freshly prepared and administrations began at 9:00 a.m. At the 20^th^ day, animals except CON group received scopolamine (3 mg/kg BW, i.p.) to induce memory impairment 30 min before a training course of Y-maze test [26]. CON group received 0.9% saline (i.p.) in the same way as mentioned above (Table 1).

### Behavioral procedures

Learning and memory abilities of the mice were tested at the end of the treatment experiment by Y-maze test as described previous [24]. Briefly, each mouse was placed at the end of one arm to adapt for 5 min without electric shock. Then electric shock made the mouse move to the safe arm, where the mouse adjusted to the situation for 30 s; and then another electric shock was switched following the sequence (ABCCAB) to compel the mouse to move to the safe arm. Such trial was repeated 35 times during a training course and repeated 10 times after 24 h during the test course. During the experiment, it would be an incorrect response if the mouse did not move to the safe arm directly within 30 s. The error times in 10 tests was taken as the learning and memory ability. The more the incorrect responses, the weaker the learning and memory ability of the mouse was.

### Determination of MDA and TAOC levels, and GSH-Px, T-SOD, MPO, TNOS, nNOS, iNOS and AchE activities

After behavioral test, all mice were anesthetized by an intraperitoneal injection of sodium thiopental (40 mg/kg BW). Blood of each mouse was obtained from the tail veins, afterwards, mice were sacrificed by cervical dislocation under anesthesia. The brain was immediately removed and washed with ice-cold normal saline. The colon was immediately dissected and its content was removed. Left brain and the colon were weighed and stored at −80°C, and then homogenized with a phosphate buffer (50 mmol/L, pH 7.0) containing 0.1 mmol/L EDTA before use. Brain homogenate was divided into two parts. One part was centrifuged at 2054 *g* for 10 min at 4°C and the supernatant was used for MDA, TAOC, GSH-Px, T-SOD, TNOS, nNOS, iNOS and AchE tests. Another part was used for determination of MPO activity directly. Blood and colon homogenate were centrifuged at 2054 *g* for 10 min at 4°C to obtain serum and colon supernatant for MDA, TAOC, GSH-Px and T-SOD tests. All parameters were determined using the respective kits according to the manufacturer's specifications.

### Quantitative real-time PCR

For nNOS, total RNA of right brain (four in each group) was isolated using Trizol reagent (15596-026, Invitrogen) according to the manufacturer's instruction, and reverse-transcribed using a RevertAid First Strand cDNA Synthesis Kit (K-1622, Fermentas) at 42°C for 30 min followed by 70°C for 5 min. Real-time polymerase chain reaction was executed with Thunderbird SYBR qPCR Mix (QPS-201, Toyobo Biologics), using the real-time thermocycler (Hangzhou Bioer Technology Co., LTD). The cycle conditions were as follows: denaturation (95°C for 60 s), cycles (40 times), renaturation (temperature declines to 58°C in 15 s), stretch (temperature increases to 72°C in 20 s, and then keeps at 72°C for 20 s). The dissociation curve of each gene was performed and analyzed using the SLAN Quantitative Real-Time PCR detection system (Shanghai Hongshi Medical Technology Co., Ltd), and the result verified the specificity of the product. Each sample was performed in triplicate, and normalized to β-actin. The relative expression levels of the genes were calculated by the 2^−ΔΔCT^ method as previously described [27]. The sequences of the primers (Invitrogen) for the genes were as follows. nNOS: forward 5′-GCTTCAGGAATATGAGGAATGG-3′, reverse 5′-TGATGGAATAGTAGCGAGGTTGT-3′; β-actin: forward 5′-CTGAGAGGGAAATCGTGCGT-3′, reverse 5′-CCACAGGATTCCATACCCAAGA-3′.

### Statistical analysis

Data were expressed as mean ± standard deviation (SD). All data were analyzed using a one-way ANOVA, followed by Duncan *post hoc test*. Statistical analyses were performed by the SPSS 16.0 software and P<0.05 was regarded as statistical significance.

## Results

### Effects of combined LSPC and LC on behavioral performance in Y-maze test

**Figure 1:**
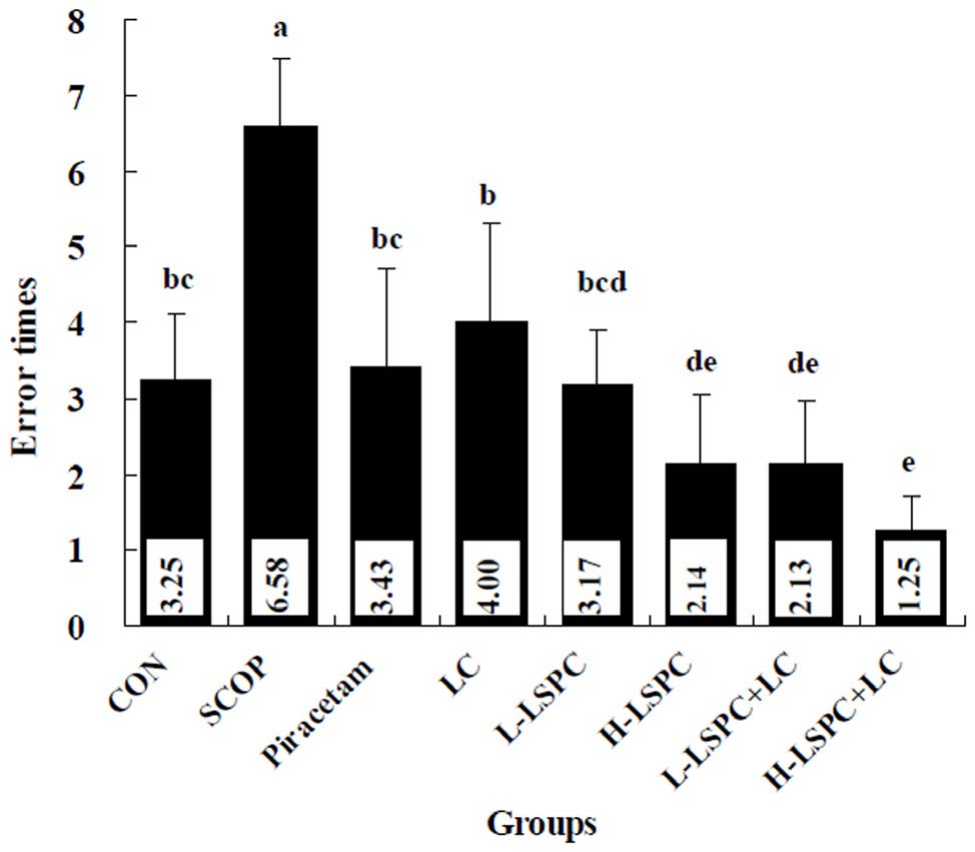
Effects of combined proanthocyanidins extracted from lotus seedpod (LSPC) and *Lactobacillus casei-01* (LC) on scopolamine-induced memory impairment in Y-maze test. Groups without any same letters above the bars signify statistically significant differences (P<0.05). CON, SCOP, Piracetam and LC is control, vehicle scopolamine control, positive drug control and *Lactobacillus casei-01* group, respectively. L-LSPC and H-LSPC is low and high dose of LSPC group, respectively. L-LSPC+LC is L-LSPC and LC combination group. H-LSPC+LC is H-LSPC and LC combination group.

Effects of combined LSPC and LC on behavioral performance in Y-maze test are shown in Fig. 1. SCOP group exhibited obviously increased error times in Y-maze test in comparison with CON group (P<0.05). However, error times were significantly reduced by treatments with Piracetam, LC, L-LSPC, H-LSPC and LSPC and LC combinations as compared to SCOP group (all P<0.05). Importantly, H-LSPC, L-LSPC+LC and H-LSPC+LC groups exhibited the decreased error times with respect to CON, Piracetam and LC groups (all P<0.05). Moreover, error times of H-LSPC+LC group were reduced by 41.59% relative to those of H-LSPC group without significant difference.

### Effects of combined LSPC and LC on brain antioxidant defense capacity and oxidative damage

MDA and TAOC levels, and activities of GSH-Px, T-SOD and MPO in brain were measured to investigate the changes of brain oxidative damage and antioxidant defense capacity. As shown in Table 2, scopolamine resulted in noticeable increases in MDA level and MPO activity, and marked declines in TAOC level, T-SOD and GSH-Px activities (all P<0.05). Compared to SCOP group, LC treatment declined MDA level, and raised TAOC level and GSH-Px activity remarkably (all P<0.05), but had no significant effect on T-SOD and MPO activities. However, treatments with Piracetam, L-LSPC, H-LSPC, L-LSPC+LC and H-LSPC+LC significantly reversed scopolamine-induced changes of MDA and TAOC levels, and T-SOD, GSH-Px and MPO activities, to the level of equal or superior to CON group. Moreover, compared to LC group, both L-LSPC and H-LSPC groups showed the obviously reduced MDA level and MPO activity, and the markedly raised activities of T-SOD and GSH-Px (all P<0.05). Importantly, MDA and TAOC levels, and T-SOD, GSH-Px and MPO activities in H-LSPC+LC group were significantly better than those in LC group (all P<0.05). H-LSPC+LC treatment exhibited higher TAOC level and GSH-Px activity than H-LSPC treatment (P<0.05 and P<0.05). Additionally, as indexes of oxidative stress, ratio of T-SOD to MDA (T-SOD/MDA) and ratio of GSH-Px to T-SOD (GSH-Px/T-SOD) were also calculated. Scopolamine led to marked declines in T-SOD/MDA and GSH-Px/T-SOD (P<0.05 and P<0.05). However, all treatments normalized GSH-Px/T-SOD. T-SOD/MDA was enhanced in all treatments groups relative to SCOP group (all P<0.05). Treatments with L-LSPC and H-LSPC+LC significantly raised T-SOD/MDA in comparison with CON group (P<0.05 and P<0.05). Effect of H-LSPC+LC treatment on improving T-SOD/MDA was markedly superior to those of both H-LSPC and LC treatments (P<0.05 and P<0.05). Furthermore, compared to Piracetam group, H-LSPC+LC group exhibited significant increases in TAOC level, GSH-Px activity and T-SOD/MDA, and a significant decrease in MPO activity (all P<0.05).

**Table 2:** Effects of combined proanthocyanidins extracted from lotus seedpod (LSPC) and *Lactobacillus casei-01* (LC) on brain malondialdehyde (MDA) and total antioxidant capacity (TAOC) levels, total superoxide dismutase (T-SOD), glutathione peroxidase (GSH-Px), myeloperoxidase (MPO) and acetylcholinesterase (AchE) activities, ratio of T-SOD to MDA (T-SOD/MDA) and ratio of GSH-Px to T-SOD (GSH-Px/T-SOD) in scopolamine-induced amnesia mice.

Group	MDA (nmol/mgprot)	T-SOD (U/mgprot)	GSH-Px (U/mgprot)	TAOC (U/mgprot)	MPO (U/g)	AchE (U/mgprot)	T-SOD/MDA	GSH-Px/T-SOD
CON	1.72±0.13^bc^	113.36±7.74^bc^	59.51±3.77^c^	1.16±0.11^bc^	0.12±0.01^bc^	0.47±0.05^b^	66.04±5.62^cd^	0.53±0.04^b^
SCOP	2.07±0.22^a^	102.72±2.31^a^	42.15±4.20^a^	0.84±0.10^a^	0.13±0.01^a^	0.59±0.07^a^	50.12±5.02^a^	0.41±0.02^a^
Piracetam	1.63±0.15^cd^	119.98±5.05^cd^	62.86±4.04^c^	1.11±0.06^b^	0.12±0.01^bc^	0.48±0.09^b^	73.83±5.30^de^	0.53±0.04^b^
LC	1.87±0.14^b^	106.46±10.79^ab^	55.59±5.10^b^	1.15±0.11^bc^	0.13±0.02^ab^	0.55±0.04^a^	56.84±2.82^b^	0.53±0.06^b^
L-LSPC	1.61±0.13^cd^	119.86±7.96^cd^	61.19±2.74^c^	1.10±0.10^b^	0.10±0.01^d^	0.44±0.05^bc^	75.01±7.42^ef^	0.51±0.03^b^
H-LSPC	1.68±0.16^cd^	118.43±8.00^cd^	60.92±3.20^c^	1.19±0.13^bc^	0.10±0.01^d^	0.47±0.04^b^	71.16±7.23^cde^	0.52±0.05^b^
L-LSPC+LC	1.60±0.21^cd^	113.89±6.55^bc^	62.46±5.12^c^	1.15±0.10^bc^	0.11±0.01^cd^	0.48±0.08^b^	72.02±10.01^cde^	0.55±0.06^b^
H-LSPC+LC	1.51±0.12^d^	124.03±3.79^d^	66.63±4.21^d^	1.32±0.11^d^	0.10±0.01^d^	0.38±0.04^c^	82.55±5.26^fg^	0.54±0.03^b^

Notes: Means in the same column with different superscript are significantly different (P<0.05), while sharing any same letters signify insignificant differences. CON, SCOP, Piracetam and LC is control, vehicle scopolamine control, positive drug control and *Lactobacillus casei-01* group, respectively. L-LSPC and H-LSPC is low and high dose of LSPC group, respectively. L-LSPC+LC is L-LSPC and LC combination group. H-LSPC+LC is H-LSPC and LC combination group.

Based on the results, LC, LSPC, and their combinations had the ability to improve brain antioxidant defense capacity and suppress oxidative damage, and the abilities of LSPC, and combined LSPC and LC were more potent than LC. LC enhanced the ameliorative effects of H-LSPC on brain antioxidant defense ability and oxidative damage.

### Effects of combined LSPC and LC on brain AchE activity

ANOVA indicated that significantly increased brain AchE activity in both SCOP and LC groups was observed when compared with CON group (P<0.05 and P<0.05) (Table 2). Brain AchE activity in Piracetam, L-LSPC, H-LSPC and the combination groups was markedly weaker than that in SCOP group (all P<0.05). Importantly, H-LSPC+LC treatment reduced brain AchE activity significantly as compared to CON, Piracetam, LC, H-LSPC and L-LSPC+LC groups (all P<0.05). LC facilitated the ameliorative effects of H-LSPC on brain cholinergic activity.

### Effects of combined LSPC and LC on serum antioxidant defense capacity and oxidative damage

As shown in Table 3, scopolamine resulted in an obvious enhancement of serum MDA level, and significant reductions of serum TAOC level, GSH-Px and T-SOD activities, and T-SOD/MDA relative to CON group (all P<0.05). LC treatment notably enhanced GSH-Px and T-SOD activities (P<0.05 and P<0.05) compared with SCOP group. Piracetam, L-LSPC, H-LSPC and L-LSPC+LC treatments normalized the variations of serum MDA and TAOC levels, GSH-Px and T-SOD activities and T-SOD/MDA induced by scopolamine. No significant differences in these five parameters were found among Piracetam, L-LSPC and H-LSPC groups. Serum TAOC level of H-LSPC group was significantly higher than that of LC group (P<0.05). L-LSPC+LC treatment showed a marked increase in T-SOD/MDA relative to both LC and L-LSPC treatments (P<0.05 and P<0.05). It was worthy of note that serum MDA, TAOC levels and GSH-Px activity as well as T-SOD/MDA in H-LSPC+LC group were better than those in CON, Piracetam, LC, L-LSPC, H-LSPC and L-LSPC+LC groups (all P<0.05). These results revealed that LC promoted the ameliorative effects of H-LSPC on serum antioxidant defense ability and oxidative damage.

**Table 3:** Effects of combined proanthocyanidins extracted from lotus seedpod (LSPC) and *Lactobacillus casei-01* (LC) on serum malondialdehyde (MDA) and total antioxidant capacity (TAOC) levels, total superoxide dismutase (T-SOD) and glutathione peroxidase (GSH-Px) activities, ratio of T-SOD to MDA (T-SOD/MDA) and ratio of GSH-Px to T-SOD (GSH-Px/T-SOD) in scopolamine-induced amnesia mice.

Group	MDA (nmol/mL)	T-SOD (U/mL)	GSH-Px (U/mL)	TAOC (U/mL)	T-SOD/MDA	GSH-Px/T-SOD
CON	24.43±9.10^bc^	196.05±15.62^bcd^	581.28±29.53^b^	5.29±0.51^bc^	8.97±2.90^bcd^	2.98±0.25
SCOP	33.87±9.12^a^	170.85±20.13^a^	529.51±33.92^a^	4.65±0.35^a^	5.40±1.64^a^	3.14±0.45
Piracetam	23.45±7.73^bc^	201.59±18.35^bcd^	604.52±17.45^b^	5.19±0.28^bc^	9.38±2.70^cd^	3.02±0.25
LC	29.22±6.63^ab^	188.01±12.95^b^	602.07±24.45^b^	5.03±0.20^ab^	6.86±2.13^ab^	3.22±0.31
L-LSPC	25.32±6.91^bc^	189.67±19.60^bc^	585.59±31.48^b^	5.18±0.21^bc^	7.80±1.44^bc^	3.13±0.46
H-LSPC	23.03±5.60^bc^	195.42±11.30^bcd^	599.62±29.08^b^	5.41±0.23^c^	8.91±2.02^bcd^	3.08±0.25
L-LSPC+LC	19.11±5.89^c^	206.80±22.87^cd^	599.66±19.67^b^	6.24±0.75^cd^	12.06±4.75^d^	2.93±0.32
H-LSPC+LC	11.54±3.28^d^	210.88±10.84^d^	627.46±22.79^c^	6.40±0.68^d^	19.38±4.49^e^	2.98±0.17

Notes: Means in the same column with different superscript are significantly different (P<0.05), while sharing any same letters signify insignificant differences. CON, SCOP, Piracetam and LC is control, vehicle scopolamine control, positive drug control and *Lactobacillus casei-01* group, respectively. L-LSPC and H-LSPC is low and high dose of LSPC group, respectively. L-LSPC+LC is L-LSPC and LC combination group. H-LSPC+LC is H-LSPC and LC combination group.

### Effects of combined LSPC and LC on colon antioxidant defense capacity and oxidative damage

As shown in Table 4, obviously raised colon MDA level, and reduced colon GSH-Px activity, T-SOD/MDA and GSH-Px/T-SOD in SCOP group were observed when compared with CON group (all P<0.05). LC treatment significantly lowered MDA level and increased T-SOD/MDA compared to SCOP group (P<0.05 and P<0.05). Moreover, treatments with Piracetam, L-LSPC, H-LSPC, L-LSPC+LC and H-LSPC+LC markedly reversed scopolamine-induced changes in colon MDA and TAOC levels, GSH-Px activity, T-SOD/MDA and GSH-Px/T-SOD, to the level of equal or superior to CON group. By comparison with SCOP group, H-LSPC+LC treatment significantly enhanced T-SOD activity (P<0.05). Furthermore, both L-LSPC and H-LSPC groups showed notable increases in TAOC level, GSH-Px activity and GSH-Px/T-SOD versus LC group (all P<0.05). Effects of H-LSPC+LC treatment on improving T-SOD and GSH-Px activities, TAOC level, T-SOD/MDA and GSH-Px/T-SOD were superior to those of LC treatment (all P<0.05), and only GSH-Px activity in H-LSPC+LC group was higher than that in H-LSPC group (P<0.05). Moreover, compared to Piracetam group, H-LSPC+LC group showed significant increases in GSH-Px activity and GSH-Px/T-SOD (P<0.05 and P<0.05). These results suggested that ameliorative effects of LSPC, and combined LSPC and LC on colon antioxidant defense ability and oxidative stress were stronger than LC. LC was helpful for H-LSPC to promote colon antioxidant defense ability.

**Table 4:** Effects of combined proanthocyanidins extracted from lotus seedpod (LSPC) and *Lactobacillus casei-01* (LC) on colon malondialdehyde (MDA) and total antioxidant capacity (TAOC) levels, total superoxide dismutase (T-SOD) and glutathione peroxidase (GSH-Px) activities, ratio of T-SOD to MDA (T-SOD/MDA) and ratio of GSH-Px to T-SOD (GSH-Px/T-SOD) in scopolamine-induced amnesia mice.

Group	MDA (nmol/mgprot)	T-SOD (U/mgprot)	GSH-Px (U/mgprot)	TAOC (U/mgprot)	T-SOD/MDA	GSH-Px/T-SOD
CON	0.74±0.10^b^	58.91±5.49^abc^	304.34±26.27^b^	0.79±0.12^ab^	81.35±15.05^bc^	5.18±0.39^bc^
SCOP	0.89±0.14^a^	55.18±3.20^a^	250.40±21.40^a^	0.65±0.17^a^	63.31±12.00^a^	4.57±0.60^a^
Piracetam	0.74±0.09^b^	58.57±2.52^abc^	305.56±14.75^b^	0.98±0.12^bc^	80.44±9.48^bc^	5.23±0.37^bc^
LC	0.75±0.16^b^	55.47±5.21^a^	269.80±35.94^a^	0.70±0.15^ab^	76.57±15.98^b^	4.89±0.66^ab^
L-LSPC	0.76±0.11^b^	58.73±5.46^abc^	345.84±42.01^cd^	0.97±0.05^c^	78.98±11.64^bc^	5.88±0.44^d^
H-LSPC	0.75±0.08^b^	58.61±3.76^abc^	333.71±45.68^bc^	1.04±0.09^c^	79.21±9.01^bc^	5.71±0.80^cd^
L-LSPC+LC	0.70±0.12^b^	57.52±3.92^ab^	320.15±27.06^bc^	0.93±0.20^bc^	84.71±16.29^bc^	5.59±0.61^cd^
H-LSPC+LC	0.69±0.12^b^	62.08±2.77^c^	365.92±43.16^d^	1.29±0.31^c^	92.19±14.27^c^	5.89±0.54^d^

Notes: Means in the same column with different superscript are significantly different (P<0.05), while sharing any same letters signify insignificant differences. CON, SCOP, Piracetam and LC is control, vehicle scopolamine control, positive drug control and *Lactobacillus casei-01* group, respectively. L-LSPC and H-LSPC is low and high dose of LSPC group, respectively. L-LSPC+LC is L-LSPC and LC combination group. H-LSPC+LC is H-LSPC and LC combination group.

### Effects of combined LSPC and LC on brain TNOS, iNOS and nNOS activities and nNOS mRNA level

As shown in Table 5, brain TNOS and nNOS activities of SCOP group were markedly increased (P<0.05 and P<0.05) relative to those of CON group. Between SCOP and LC groups, there were no significant differences in brain TNOS, iNOS and nNOS activities. However, treatments with Piracetam, L-LSPC, H-LSPC, L-LSPC+LC and H-LSPC+LC normalized the raised TNOS and nNOS activities induced by scopolamine, and no remarkable difference was found among these groups. Importantly, brain iNOS activity was notable diminished by H-LSPC+LC treatment compared with SCOP, Piracetam, L-LSPC and L-LSPC+LC groups (all P<0.05).

**Table 5:** Effects of combined proanthocyanidins extracted from lotus seedpod (LSPC) and *Lactobacillus casei-01* (LC) on brain total nitric oxide synthase (TNOS), nitric oxide synthase (iNOS) and neural nitric oxide synthase (nNOS) activities in scopolamine-induced amnesia mice.

Group	TNOS (U/mgprot)	iNOS (U/mgprot)	nNOS (U/mgprot)
CON	0.48±0.06^b^	0.14±0.02^ab^	0.34±0.08^b^
SCOP	0.60±0.05^a^	0.15±0.02^a^	0.45±0.05^a^
Piracetam	0.48±0.04^b^	0.15±0.02^a^	0.35±0.07^b^
LC	0.60±0.08^a^	0.14±0.02^ab^	0.46±0.07^a^
L-LSPC	0.48±0.06^b^	0.15±0.01^a^	0.34±0.07^b^
H-LSPC	0.45±0.09^b^	0.14±0.04^ab^	0.30±0.09^b^
L-LSPC+LC	0.50±0.04^b^	0.16±0.02^a^	0.35±0.04^b^
H-LSPC+LC	0.47±0.06^b^	0.12±0.03^b^	0.36±0.06^b^

Notes: Means in the same column with different superscript are significantly different (P<0.05), while sharing any same letters signify insignificant differences. CON, SCOP, Piracetam and LC is control, vehicle scopolamine control, positive drug control and *Lactobacillus casei-01* group, respectively. L-LSPC and H-LSPC is low and high dose of LSPC group, respectively. L-LSPC+LC is L-LSPC and LC combination group. H-LSPC+LC is H-LSPC and LC combination group.

**Figure 2:**
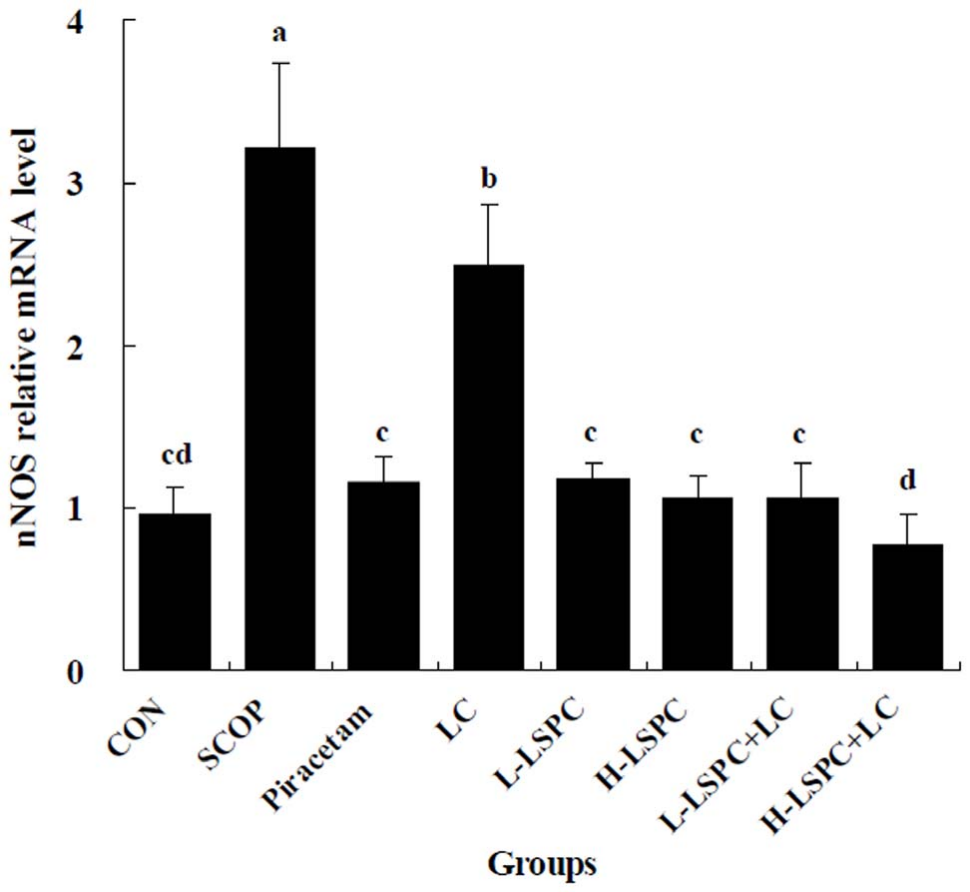
Effects of combined proanthocyanidins extracted from lotus seedpod (LSPC) and *Lactobacillus casei-01* (LC) on the mRNA level of neural nitric oxide synthase (nNOS). Groups without any same letters above the bars signify statistically significant differences (P<0.05). CON, SCOP, Piracetam and LC is control, vehicle scopolamine control, positive drug control and *Lactobacillus casei-01* group, respectively. L-LSPC and H-LSPC is low and high dose of LSPC group, respectively. L-LSPC+LC is L-LSPC and LC combination group. H-LSPC+LC is H-LSPC and LC combination group.

The variations in brain mRNA level of nNOS were parallel to the alterations in brain nNOS activity in all the experimental groups. As shown in Fig. 2, SCOP group showed significantly higher nNOS mRNA level than CON group. LC group revealed a significant decrease in nNOS mRNA level relative to SCOP group (P<0.05), but showed significantly higher nNOS mRNA level than CON group (P<0.05). However, nNOS mRNA level in Piracetam, L-LSPC, H-LSPC, L-LSPC+LC and H-LSPC+LC groups was remarkably lower than that in both SCOP and LC groups (all P<0.05), and comparative with that in CON group. Furthermore, H-LSPC+LC group exhibited a significant decline in nNOS mRNA level relative to Piracetam, LC, L-LSPC, H-LSPC and L-LSPC+LC groups (all P<0.05).

## Discussion

The present study provides initial evidence that supports the hypothesis that LC facilitates the ameliorative effects of LSPC on learning and memory impairment in scopolamine-induced amnesia mice. In our study, H-LSPC+LC group had better behavioral performance in Y-maze test than H-LSPC and LC groups. Moreover, LC promoted the memory-enhancing effect of LSPC by improving antioxidant defense ability of brain, serum and colon, ameliorating brain cholinergic activity, and suppressing oxidative damage of serum and brain as well as brain nNOS mRNA level.

Animals with scopolamine-induced memory impairment have been generally employed to appraise the possible memory-improving activity of herbal and other agents [24], [28]. In this study, scopolamine resulted in elevated error times in Y-maze test compared with CON group, which verified the serious memory deterioration in mice [28]. Piracetam, a clinic medicine, has shown the memory-enhancing effect in many animal model systems [29]. Compared with Piracetam group, LC and L-LSPC groups showed comparative effects on attenuating scopolamine-induced memory impairment in Y-maze test. However, H-LSPC, and LSPC and LC combinations exhibited stronger memory-enhancing effects than piracetam, which was suggested by markedly decreased error times of these groups compared to Piracetam group. The memory-enhancing effects of LC and LSPC were consistent with previous studies [6], [13], [14], [15], [22]. Noteworthily, error times of H-LSPC+LC group were reduced by 41.59% and 68.75% relative to those of H-LSPC and LC groups respectively, which indicated that LC was potentially helpful for H-LSPC to attenuate scopolamine-induced memory impairment.

Previous researches have found that learning and memory impairment is closely associated with oxidative stress [14], [15], [30]. As a muscarinic receptor antagonist, scopolamine results in memory deficits in animals by leading to cholinergic neurotransmission deficits and oxidative damage [28], [31], [32]. In this study, scopolamine resulted in noticeable declines in TAOC level, T-SOD and GSH-Px activities, T-SOD/MDA of brain and serum, and brain GSH-Px/T-SOD, and obvious increases in MDA level of brain and serum, and brain MPO activity. It is well known that TAOC reflects the capacity of nonenzymatic antioxidant defense system, and GSH-Px and SOD are two important enzymes of antioxidant defense system to eliminate reactive oxygen species (ROS) [33]. MDA, a byproduct of lipid peroxidation, is considered as the biomarker of oxidative stress [34]. The increases in GSH-Px/T-SOD and T-SOD/MDA indicate that more antioxidant enzymes are activated and ROS are efficiently scavenged by them, and thus oxidative damage is decreased [35], [36]. The results indicated that marked decreases in antioxidant defense ability and increases in oxidative damage of mice brain and serum were closely associated with scopolamine-induced memory deficits [28], [32]. LC treatment brought a certain increase in antioxidant defense ability of brain and serum, which may be due to the antioxidant abilities of *Lactobacilli* strains [37], [38]. LC treatment only significantly reversed raised MDA level of brain, revealing the weak effect of LC on inhibiting oxidative damage. Moreover, LSPC and LSPC and LC combinations reversed scopolamine-induced variations of brain and serum, verifying that enhancement of antioxidant defense ability and inhibition of oxidative damage were effective ways to improving memory [14], [30]. Previous studies have demonstrated that LSPC have a potent antioxidant activity *in vitro* [12], and an inhibitory effect on oxidative stress in brain and serum [14], [15]. Especially, LSPC groups showed comparative MDA level and T-SOD/MDA of brain and serum with Piracetam group, and remarkably lower MPO activity of brain than Piracetam group, which prompted that LSPC were the effective inhibitor of oxidative damage. Noteworthily, effects of H-LSPC+LC treatment on lowering serum MDA level and raising T-SOD/MDA, TAOC level and GSH-Px activity of brain and serum were stronger than those of Piracetam, H-LSPC and LC treatments, suggesting that LC facilitated the ameliorative effects of H-LSPC on antioxidant defense ability and oxidative damage of brain and serum.

Learning and memory impairment is associated with gut dysfunction [1], [5], [6]. Oxidative damage of colonic mucosa is one of main symptoms of intestinal dysfunction in F344 rats, and inhibition of oxidative damage is an important approach to modulate intestinal function and carcinogenesis [17]. This study measured colon MDA and TAOC levels, and activities of T-SOD and GSH-Px to evaluate the changes of gut function. Obviously raised MDA level, and lessened GSH-Px activity, T-SOD/MDA and GSH-Px/T-SOD in colon of SCOP mice revealed the increases of oxidative damage and the decreases of antioxidant defense ability in colon, and further indicated mild colon dysfunction induced by scopolamine. Previous studies have reported that *Lactobacilli* strains have antioxidant abilities [37], [38], and can modulate gut function [1], [6]. In this study, LC treatment showed the effective inhibition of colon oxidative damage, as indicated by markedly diminished MDA level and enhanced T-SOD/MDA. Thus, inhibition of oxidative damage was an approach to modulate gut function [17], and the antioxidant ability of LC may partly contribute to the amelioration of gut dysfunction. This study showed that LSPC, and LSPC and LC combinations had potent abilities of suppressing oxidative damage and improving antioxidant defense of colon. Importantly, GSH-Px activity of H-LSPC+LC group was superior to that of Piracetam, LC and H-LSPC groups, suggesting that LC facilitated the ameliorative effects of H-LSPC on antioxidant defense ability of colon. These results also indicated that improving gut dysfunction may contribute to the amelioration of learning and memory ability [1], [5], [6].

Base on the above results, LC facilitated the ameliorative effects of H-LSPC on memory impairment by improving antioxidant defense ability of brain, serum and colon, and inhibiting oxidative damage of serum and brain. LSPC had a potent antioxidant activity [12], and parts of monomers, dimers, and trimers of proanthocyanidins in LSPC were absorbed to blood and then distributed into the brain to exert the antioxidant activity [39]–[41], therefore, oxidative damage and antioxidant defense ability of brain and serum were ameliorated. Previous researches proved the gut-regulation effect and antioxidant ability of *Lactobacilli* strains [37], [38], thus in this study, LC showed a certain capacity of inhibiting oxidative damage and enhancing antioxidant defense ability. Most ingredients of LSPC were not absorbed directly and then accumulated in colon [18], [19]. In colon, LSPC were metabolized by the colonic microbiota and generated phenolic acids, oligomeric proanthocyanidins and their isomers, and conjugated lactones [42], [43], which showed stronger physiological and biological activities than their parent proanthocyanidins [19], [44]. Proanthocyanidins metabolites by biotransformation of LC *in vitro* showed more potent antioxidant ability than their parent proanthocyanidins [23], thus the metabolites of the combination groups exerted stronger antioxidant ability than LC and LSPC groups *in vivo*. Additionally, proanthocyanidins metabolites in colon kept the growth of probiotics such as *Lactobacillus* and inhibited the growth of pathogenic bacteria [20]. Consequently, in this study, the metabolites of the combination groups exerted stronger effects on improving gut function by modulating gut bacteria and decreasing oxidative damage. Ultimately, LSPC and LC combination exhibited a potent effect on ameliorating scopolamine-induced memory impairment.

## Conclusions

In conclusion, LC, LSPC, and LSPC and LC combinations exhibited the ameliorative effects on scopolamine-induced memory impairment in mice. The ameliorative effects of LSPC, and LSPC and LC combinations were more effective than LC. The mechanisms involved in improving memory deficits of LSPC and LC combinations were associated with the improvement of antioxidant defense ability of brain, serum and colon, inhibition of oxidative damage of brain, serum and colon, suppression of brain nNOS activity and mRNA level, and amelioration of brain cholinergic activity. LC promoted the memory-enhancing effect of LSPC by improving antioxidant defense ability of brain, serum and colon, ameliorating brain cholinergic activity, and suppressing oxidative damage of serum and brain as well as brain nNOS mRNA level. These findings suggest LSPC and LC combination may provide a viable therapy in the treatment of memory impairment in aging process and some related diseases such as AD.
